# Xenon for tunnelling analysis of the efflux pump component OprN

**DOI:** 10.1371/journal.pone.0184045

**Published:** 2017-09-08

**Authors:** Yvette Véronique Ntsogo Enguéné, Gilles Phan, Cyril Garnier, Arnaud Ducruix, Thierry Prangé, Isabelle Broutin

**Affiliations:** 1 Laboratoire de Cristallographie et RMN Biologiques (UMR 8015, CNRS), Faculté de Pharmacie, Université Paris Descartes, USPC, Paris, France; 2 Laboratoire de Biologie Physico-Chimique des Protéines Membranaires (UMR 7099, CNRS, Université Paris Diderot) Institut de Biologie Physico-Chimique, Paris, France; Centre National de la Recherche Scientifique, Aix-Marseille Université, FRANCE

## Abstract

Tripartite efflux pumps are among the main actors responsible for antibiotics resistance in Gram-negative bacteria. In the last two decades, structural studies gave crucial information about the assembly interfaces and the mechanistic motions. Thus rigidifying the assembly seems to be an interesting way to hamper the drug efflux. In this context, xenon is a suitable probe for checking whether small ligands could act as conformational lockers by targeting hydrophobic cavities. Here we focus on OprN, the outer membrane channel of the MexEF efflux pump from Pseudomonas aeruginosa. After exposing OprN crystals to xenon gas pressure, 14 binding sites were observed using X-ray crystallography. These binding sites were unambiguously characterized in hydrophobic cavities of OprN. The major site is observed in the sensitive iris-like region gating the channel at the periplasmic side, built by the three key-residues Leu 405, Asp 109, and Arg 412. This arrangement defines along the tunnel axis a strong hydrophobic/polar gradient able to enhance the passive efflux mechanism of OprN. The other xenon atoms reveal strategic hydrophobic regions of the channel scaffold to target, with the aim to freeze the dynamic movements responsible of the open/close conformational equilibrium in OprN.

## Introduction

**Fig 1:**
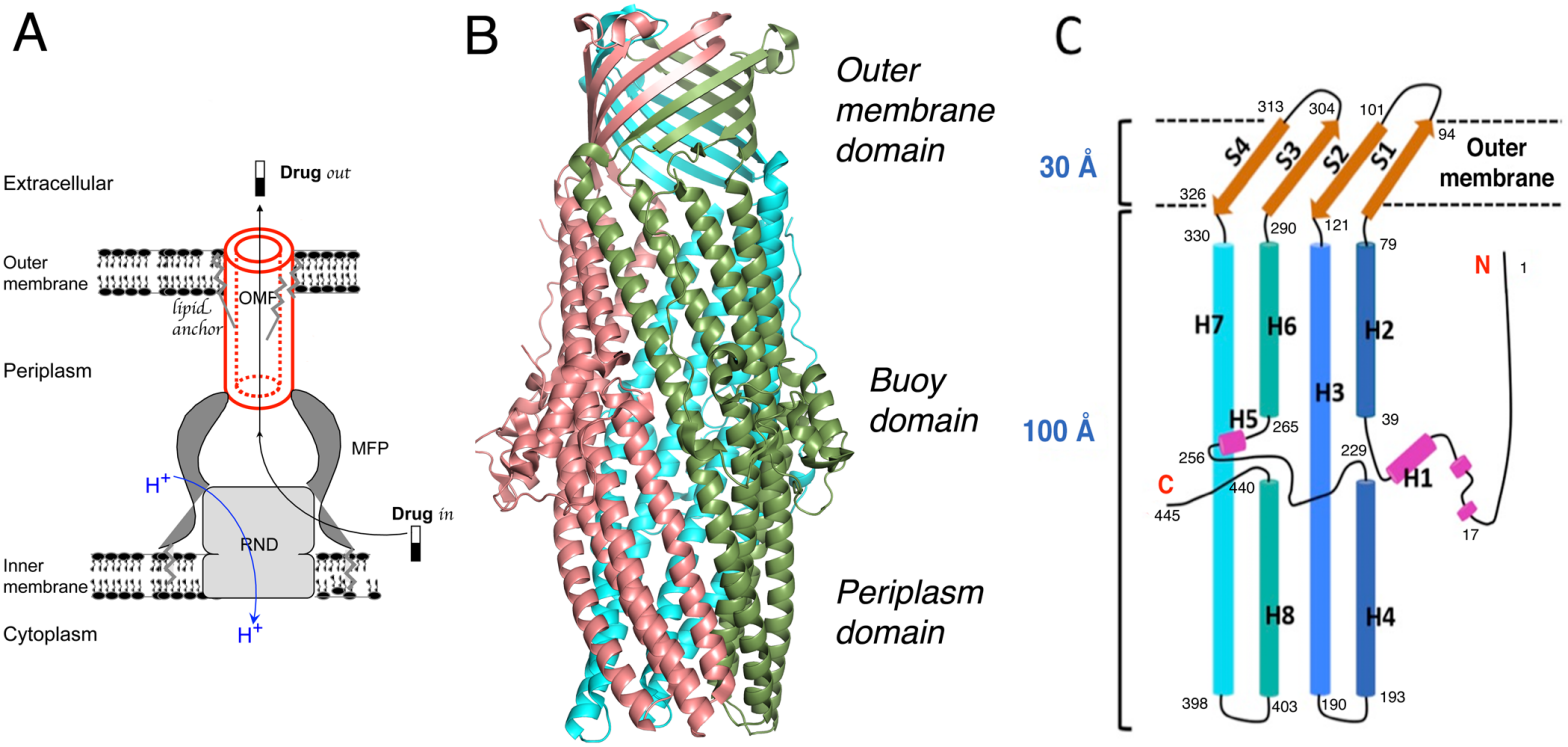
Views of the tripartite assembly of an efflux pump. **A**: The schematic reconstitution complex supported by microscopy images on the MexAB-OprM in nanodiscs and by the chimeric AcrAB-TolC-AcrZ complex [9]. The oligomerization state is 3:6:3 for the three components, respectively. The RND part uses the proton motive force and is responsible of the active counter transport of the drug. **B**: The overall trimer assembly of OprN (the OMF component -in red at left- of the MexEF-OprN assembly). **C**: Topology of secondary structures of the OprN monomer with β-strands S1 to S4 and α-helices H1 to H7.

Pseudomonas aeruginosa is an opportunistic bacterium frequently found in nosocomial infections and chronic lung infections of Cystic Fibrosis patients. Nowadays it is a serious challenge in hospital environments because of the appearance of multidrug resistant strains [1, 2]. The intrinsic and acquired resistance phenotypes are highly linked to the rapid and active efflux of the drugs by a membrane protein complex made of three protein partners [3, 4], called the efflux pump system. In P. aeruginosa, at least 12 systems have been biochemically characterized [5], such as MexA-MexB-OprM (or commonly named MexAB-OprM) which is constitutively expressed, and MexE-MexF-OprN (or MexEF-OprN), which expression is triggered under antibiotic pressure [6, 7]. In MexAB-OprM, MexB belongs to the RND (Resistance Nodulation cell Division) membrane transporter superfamily, which actively pump out the drug at the inner-membrane; MexA is a periplasmic component (MFP or Membrane Fusion Protein) that stabilizes the whole system, and OprM is a tunnel-like component which allows the routing of the drug through the outer-membrane following a passive mechanism (Fig 1 left, for a review see [8]). Although a fully assemble efflux pump was recently described by single-particle electronic microscopy [9, 10], the selective export mechanism of the drug by outer-membrane channels remains elusive.

There is a large amount of data regarding the structure of the homotrimeric outer-membrane channel. Mutagenesis [11], X ray diffraction structures [12–14], normal mode analysis [13] and in silico dynamics [15], have converged toward a plausible mechanism of channel gating where an iris-like motion of the α-helical coiled-coil domain [14] is combined with a twist/stretch mechanism [13]. Systematic mutagenesis on one hand, and salt-bridge analysis on the other hand, gave additional information on residues involved in the drugs efflux [8, 11]. In addition, cobalt hexamine was found to inhibit the homologous channel TolC from E. Coli and the X-ray structure revealed that the inhibition took place at the conserved residues Asp 374 / Asp 371 [16]. Moreover, molecular dynamics simulations supported a special function of residues Val 408 and Asp 416 in OprM gating [15].

To gain more information regarding the dynamics of the OMF tunnelling mechanism, we focused our interest on OprN, the OMF protein associated with the MexEF complex within P. aeruginosa (Fig 1 right). This efflux pump is implicated in the resistance to fluoroquinolones, the most used antibiotic class. Although OprN has only a moderate degree of homology with OprM (31.4%), its structure was solved by Molecular Replacement with OprM X-ray structure [17, 18]. To determine whether small hydrophobic ligands could block the iris-like gate or freeze the channel conformation by targeting non-polar cavities, xenon appeared to be an ideal probe [19–23].

## Materials and methods

### OprN purification and crystallization

The oprN gene was cloned into the plasmid pBAD-33 with a 6-His tag at the C-terminus. The purification protocol was already described [18] but some modifications were introduced as follows. After transformation in C43-ΔacrB strain, the culture was performed at 37°C until an OD_600_ = 0.6, then cooled-down at 4°C during 30 minutes before induction with a final concentration of 0.02% L-arabinose. Culture was continued overnight at 20°C. The cell pellets were resuspended in 20 mM Tris–HCl pH 8, 150 mM NaCl, lysed by two runs at 2.4 kbar in a Cell-disruptor (Constant Systems Ltd), and centrifuged for 30 minutes at 8000 g. The supernatant was then centrifuged at 145000 g for 1 hour at 4°C to isolate the membranes. The pellet was dissolved in the same buffer with 0.5% (w:w) N-Lauryl-Sarcosine to specifically solubilize the inner membrane and centrifuged 1 hour at 145000 g leading to a pellet containing the outer membrane only. It was solubilized overnight at 4°C in the buffer with 1.5% (w:v) DDM (n-Dodecyl-β-D-Maltopyranoside) and the detergent was exchanged with 0.9% (w:v) β-OG (Octyl-β-D-Glucopyranoside) during the Ni-NTA purification step (HisTrap agarose column, GE Healthcare). A last size exclusion chromatography step was performed on a Superose 6 column (GE Healthcare) in 20 mM Tris-HCl pH 8, 150 mM NaCl and 0.9% (w:v) β-OG. The purified protein was concentrated to 12 mg/mL for crystallization. Needle crystals were obtained within a week at 18°C by mixing equal volumes of protein with 100 mM sodium acetate, pH 5.4, 0.8–1.1 M sodium formate and 9–11% PEG 4000 (w/v). Finally 30% glycerol was added to the crystallization drop as cryo-protectant before flash-cooling in liquid nitrogen.

### X-ray data collection and integration

Diffraction data for OprN native structure (OprN-wt) were recorded at the ESRF synchrotron (Grenoble, France) on ID23-2 beamline at a wavelength of 0.873 Å, with a Pilatus3-2M detector (Dectris). Xenon bound derivative (OprN-Xe) crystals were obtained from OprN-wt crystals incubated with a pressure of 2 MPa (20 bar) of xenon. After about 2–4 minutes, the pressure was released and the crystal immediately plunged in liquid nitrogen for quenching. Several crystals were selected for xenon exposure according to the above protocol and the best diffracting crystal was recorded at the ESRF, on beamline ID29. We optimally selected a wavelength of 1.77 Å as to maximize the anomalous signal of xenon (the Δf" contribution is estimated greater than 9 electrons) and limit absorption of X-rays that becomes at low energy a serious limiting factor on intensities (and hence on data accuracy). The detector in use was a Pilatus3-6M (Dectris). The two OprN-wt and OprN-Xe data sets were indexed and merged with the XDS program [24] in the I4 tetragonal space group.

Data sets were scaled and merged with the POINTLESS/AIMLESS programs [25], part of the CCP4 suite [26, 27] and converted to structure factors with the CTRUNCATE program [28]. The two data sets showed low quality as deduced from their R factors agreement, especially in the case of OprN-Xe crystals because of low I/s(I) ratios resulting from the high absorption of diffracting spots at the selected wavelength. In that latter case, a full sphere of diffraction was recorded with a high redundancy with the aim to minimize errors by averaging the weak signals. This strategy was strengthened by Rpim values being always and systematically low, promising a fruitful exploitation of the diffraction data. The statistics of the best data collections are reported in Table 1 for the two OprN-wt and OprN-Xe structures.

**Table 1:** Data recording statistics and refinements (T = 100 K) for the OprN-wt and the OprN-Xe complex. Both are isomorphous, in the tetragonal I4 space group (Data from the highest resolution bin are in parentheses).

	OprN-wt	OprN-Xe
**Data collection and integration statistics**		
Synchrotron beam line	ID23-2	ID29
Wavelength (Å)	0.873	1.771
Cell parameters (Å) a = b; c	257.65; 81.51	256.41; 81.39
Matthews coefficient (Å^3^/Da)	2.32	2.29
Percentage of solvent (%)	47.04	46.36
Resolution limits (Å)	182.2–2.29	181.0–2.50
	(2.34–2.29)	(2.60–2.50)
No of measured reflections	584 998	612 875
No of unique reflections	119 774	91 716
No of observed reflections	113 747	86 977
Rmerge (%)^a^	13.3 (67.0)	19.2 (67.2)
Rpim (%)^a^	7.3 (42.0)	7.4 (32.0)
CC1/2 (%)^b^	98.9 (73.2)	97.7 (80.9)
Completeness (%)	97.6 (96.9)	99.5 (98.2)
Redundancy	4.9 (4.5)	6.7 (6.2)
I/σ(I)	8.01 (1.76)	4.50 (1.42)
Refinement statistics		
R_work_/R_free_ (% / %)	18.55 / 22.53	19.45 / 23.80
No of used reflections	113 747	86 977
No of refined atoms	11 430	10 974
No of amino-acids	1 341	1 341
No of water molecules	684	550
No of ions	2	2
No of alternates positions	11	10
No of additives	3 detergents	4 detergents
	1 formate	17 xenons
rms bonds deviation (Å)	0.02	0.02
rms angle deviation (°)	2.074	2.046
Overall thermal factor (Å^2^)	35.64	31.09
Average thermal B factors		
Main chain A / all atoms	32.92 / 36.15	29.14 / 31.20
Main chain B / all atoms	31.86 / 35.2	28.45 / 30.48
Main chain C / all atoms	32.61 / 35.40	29.57 / 31.19
Ramachandran diagram		
Preferred (%)	98.07	97.72
allowed (%)	1.78	1.60
poor (%)	0.15	0.68
PDB accession number	5iuy	5nsw

^a^ Rmerge is the standard agreement factor = ∑_H_∑_j_ (|I_H,j_−<I_H_>|) / ∑_H_∑_j_ (I_H,j_) and Rpim is the precision-indicating merging R-factor = ∑_H_√(1/(n−1)∑_j_ (|I_H,j_−<I_H_>|) / ∑_H_∑_j_ (I_H,j_). The Rmerge of OprN-Xe was always rather high but trusting on the Rpim was always the guideline for data quality.

^b^ from XDS statistics

### Structure determinations

Initial phases of the OprN-wt structure were determined by molecular replacement (BALBES pipeline, [29]) using the 3d5k OprM structure from the PDB [13] as the starting model. The OprN-wt model was then reconstructed and refined independently of the 5azp OprN model deposited in the meantime at the PDB [17]. In the OprN-Xe structure, the xenon atoms were located from anomalous difference Fourier maps ΔΔρ_an_ calculated with coefficients: $\Delta_{ano}^{\pm }\left(H\right).\text{exp}\left[i\left(\phi \left(H\right)-\frac{\pi }{2}\right)\right]$ where $\Delta_{ano}^{\pm }\left(H\right)$ are Bijvoet pair differences of structure factor moduli of OprN-Xe and *ϕ*(*H*) the phases of the refined OprN-wt structure.

### Structure refinements

Refinements of both the native OprN-wt and the xenon complex OprN-Xe structures were performed using the REFMAC5 program [30, 31] with individual isotropic factors. Standard constraints were used throughout on distances, angles, planes and isotropic factors. Refined models were checked using the Coot graphic program [32] for rebuilding’s and water/ligands localization. In the OprN-Xe structure, special care was taken to correctly assign xenon occupancy factors as they are strongly correlated with their thermal factors within the refinement steps. After each round of REFMAC5 refinement, an omit-map was calculated with i) xenon atoms plus one sulphur atom of a methionine in each subunit (used as internal reference) subtracted from the model, and ii) the refined phases. Xenon occupancies were then calibrated with the omitted sulfur atoms, and a new round of REFMAC5 refinement was restarted. After two cycles of refinements/omit-map, the occupancy factors were found at a reasonably stable level and definitively assigned. In the last round of refinements, the thermal factors of xenon atoms were allowed to refine freely (not constrained).

All the statistics regarding the refined models are also reported in Table 1. The coordinates and structure factors of OprN-wt and OprN-Xe were deposited in the PDB (accession numbers 5iuy and 5nsw, respectively). All figures were drawn with Pymol [33]. Distances between Xenon and amino acid's bound atoms are in the range of 3.6–4.7 Å, the complete list of the xenon sites and distances are reported in the Supporting Information (S1 and S2 Tables).

### Energy profile analyses

A Fortran program was written to approximate the non-bonded energy of a xenon probe rolling along the central axis of the tunnel. The Lennard-Jones parameters and formulation of the non-bonded energy were taken from the CHARMM22 forcefield for the C, N, O, and S atoms [34]. Those of xenon were derived from literature, and combined Xe-C, Xe-O, Xe-N, and Xe-S interaction parameters were obtained from the Lorentz-Berthelot combining rules. A cut-off of 7 Å was applied to the xenon atom interactions.

## Results

### Description and comparison of the OprN structures

As a representative member of the OMF family, OprN structure shows two main domains: a channel of 100 Å long located in the periplasm, also known as the α-domain, and a β-barrel of 30 Å long inserted in the outer membrane. The assembly of three monomers results in a long and wide tunnel responsible for the transport of drug out of the bacteria. In addition, an equatorial domain called the buoy domain, mainly composed of α-helices and flexible loops is located in the middle of the α-domain (Fig 1B). Surprisingly, this buoy domain is the main structural feature that differs among the OMFs proteins, both in structure and sequence. For instance, there is 31% of overall sequence identity between OprM and OprN, but only 23% for the buoy domain (structural alignments of OMFs are given in S1 Fig. An internal diameter of 35 Å is measured along the channel between the b-barrel and the buoy. The protein channel is closed by a lid of three β-turns at the extracellular side and occluded by a constriction of three a-coiled-coils at the periplasmic side, which gating mechanism is probably ruled by an iris-like movement [14].

OprN-wt and OprN-Xe structures perfectly match each other with a Cα rms deviation of 0.21 Å over 1352 atoms of the trimer, suggesting an overall protein fold unaffected by the xenon interaction. At the time we solved our OprN structure, another one appeared in the literature [17] solved in the same space group I4 (PDB code 5azp). The Cα rms deviation between 5azp and our structure is about 0.77 Å. The main differences are limited to the C-terminus orientation as well as the distribution of the detergent and additive molecules around the trimer. In 5azp, all the β-octyl glucoside (β-OG) molecules gather around the β-barrel domain, whereas in our OprN-wt structure, three β-OG were found at the β-barrel domain and one just above the buoy domain. The latter is trapped between the C-terminus, the α-helices H3 and H7 of one subunit, and the a-coiled-coil tips of another subunit (Fig 1C and S2 Fig). Although this β-OG suggests a new binding site for a glucose derivative molecule on the channel of OprN, the crystal packing may have contributed to the stabilisation of this interaction (S2 Fig).

At the N-terminus Cys residue, it was possible to construct at each monomer the acyl group of the post-translational palmitoylation. Although difficult to observe due to some disorder, 4 to 10 carbons of the aliphatic chains of the different palmitoyl were built. We also observed a dual acylation at the same modified cysteine, an unusual post-translational lipidation in the OMF family. In addition, a nickel cation complexed by the His 449 of the His-tag, Glu 139, and two water molecules, was located in the electron density map (S2A Fig).

### The xenon binding sites

**Fig 2:**
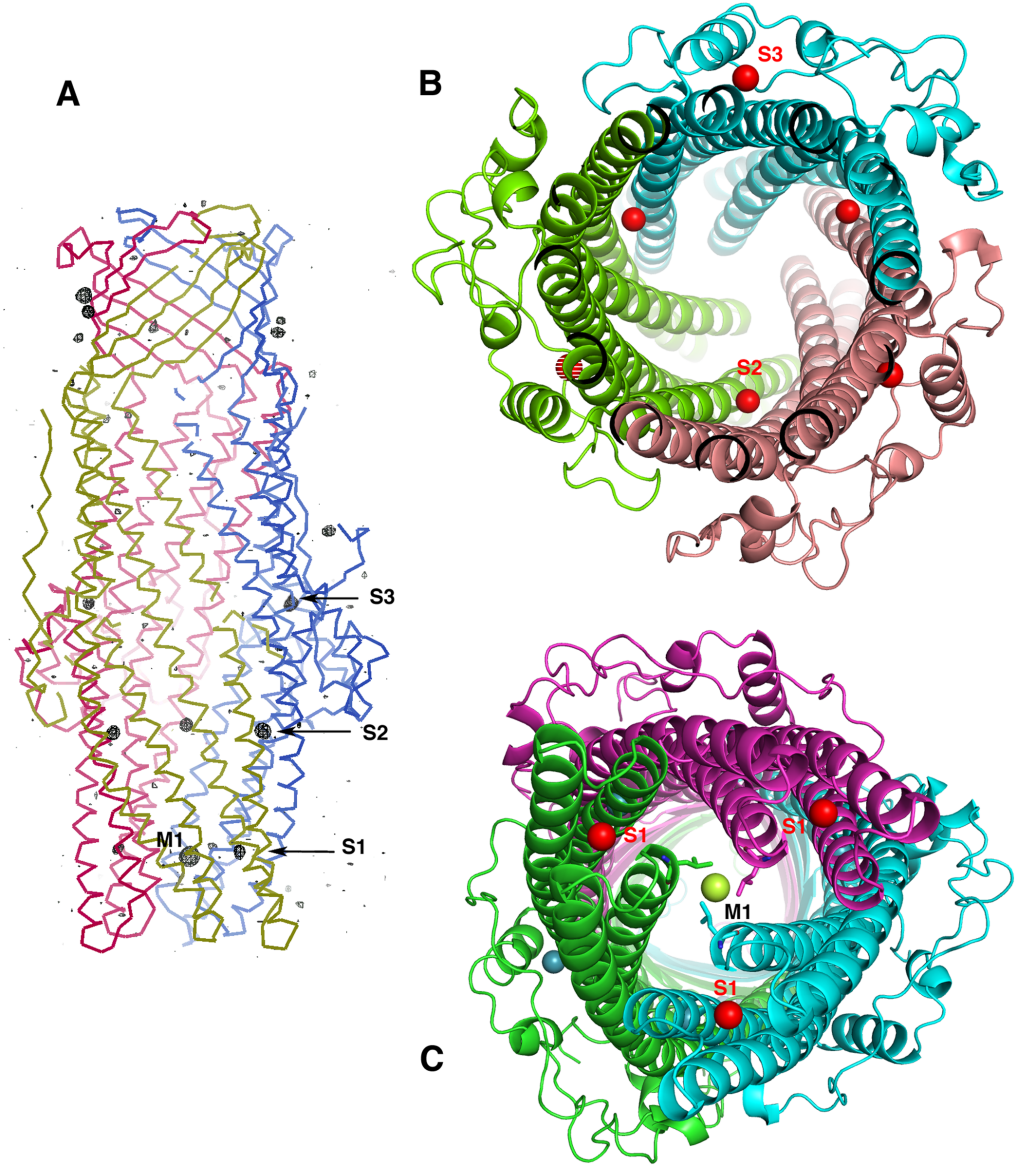
**A**: The anomalous map—in black tracing and contour at 3.5 σ.—superimposed to the Cα tracing of the OprN structure. The three S1 to S3 sites are labelled. On top of the structure, outside of the β-barrel, four more xenon atoms are present around the β-barrel domain, in a fully hydrophobic environment. **B**: (view from the extra cellular side) The two inter and intra-subunit S2 and S3 sites in the helical α-domain (xenon as red spheres) viewed along the central channel of the trimer. **C**: (view from the periplasmic side) The M1 site locked by the three equivalent Leu 405, viewed along the central channel of OprN-Xe structure at the periplasmic entrance. The S1 secondary sites are located at the same level of the main M1 site in a plane perpendicular to the tunnel. S1 xenon atoms are in red, M1 xenon in lime green. The three monomers building the cylindrical scaffold are in three different colours.

According to the anomalous map (Fig 2A), 18 peaks were located in OprN-Xe electron density. Among these peaks, 17 were identified as xenon atoms, according to their high hydrophobic environment, and one was attributed to an opportunistic nickel cation coordinated by the His-tag in subunit C (S2A Fig). Regarding xenon atoms, only one site is fully occupied, another shows a very faint signature (~10% occupancy). All the other xenon sites display partial occupancies in the range 20–40%.

### The xenon binding sites in the α-domain

The main feature of the OprN-Xe structure is a site of xenon with 100% occupancy, named M1. This site is located on the non-crystallographic three-fold axis running along the channel at the periplasmic extremity of OprN. The xenon is trapped in sandwich between the three symmetry-related Leu 405 forming a tight hydrophobic constriction of the channel at that extremity (Fig 2C). The xenon cavity is lined on the other side by the three symmetry-related Asp 409, part of a key circular salt bridge with Arg 412, two residues also highly conserved in OprM (Asp 416—Arg 419, respectively).

**Fig 3:**
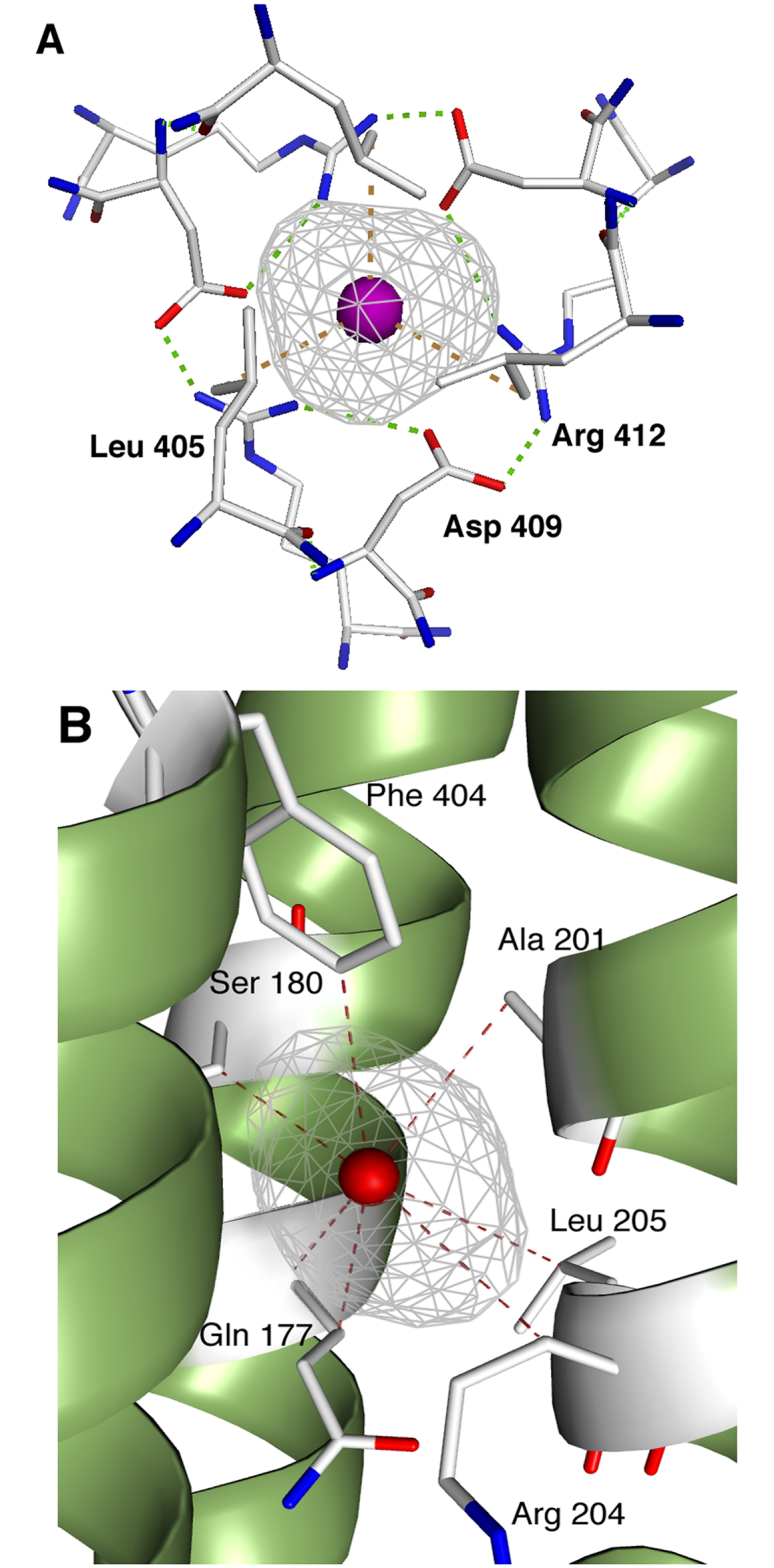
**A**: Details of the xenon's cavity M1 viewed along the tunnel. The cavity (in mesh) is built by the three hydrophobic Leu 405 side chains on one edge, and the circular salt bridge system [.. -> Asp 409a—Arg 412b → Asp 409b—Arg 412c → Asp 409c—Arg 412a →..] on the other. Hydrophobic contacts to Leu are in brown, hydrogen bonds between Glu and Arg are in green. **B**: Residues building the site S1, at the same level as M1. Note that the hydrophobic contacts towards polar residues like Gln or Arg, displace the polar part of their side chains as to keep the overall hydrophobic character of the cavity. Xenon atoms are represented as spheres with arbitrary radius.

Among the other sites, 8 are localized in the α-domain and the buoy domain in three equivalent cavities of each monomer, thus accounting for three sites termed S1 to S3 (Fig 2). In the last S3 site, only two xenon atoms are clearly visible in the anomalous map, with intensities greater than 4 σ. The third position corresponds to a small peak, at the level of the map average signal (< 1 σ). During the refinement steps, this signal was seriously degraded and further not observed in 2Fo-Fc electron density maps calculated with the refined phases (these maps are represented in S3–S5 Figs of the Supporting information). This site was thus considered void of xenon. The secondary sites S1 to S3 are all within the α-domain, either intra-subunit (S1, S3) or inter-subunit (S2). In the first S1 site, three xenon atoms are located, at the vicinity of site M1 (Fig 2C) in a specific pocket with equivalent environment and built at the core of three of the four helices bundle in each monomer (Fig 2C). At the exception of Glu 411, presenting an oxygen atom at close distance of each xenon (although larger than 3.1 Å), the environment of S1 remains as expected mostly hydrophobic, with contacts to Leu 205, Leu 408, Phe 404, together with aliphatic side chains of Ser 180, Arg 181, Arg 204 and Gln 177 (Fig 3).

The secondary site S2 also includes three xenon atoms in a similar hydrophobic environment. It is located right below the buoy, at the interface of two subunits on the inner rim of the central tunnel. The three xenon atoms are caged by the aliphatic part of the Glu 427, Arg 375, Lys 371 side chains, from one subunit, and Gln 214, Pro 213, Ala 217 from another one (Fig 2B).

The last xenon atoms (sites S3) are located on the external face of the channel, above the buoy. They appear at the level of the junction between the two helices H2 and H4 parallel to the long one, H3, which extends along the whole periplasmic domain, in the most non-symmetric part of the trimer assembly. The first xenon (S3c site) is close to the β-OG molecule in the vicinity of the C-monomer His-tag (S2 Fig). The second xenon (S3b site) lies at the same position in molecule B, despite the absence of β-OG in this monomer. The third xenon (S3a site) is only present at a low occupancy, probably less than 5%, as deduced from its weak anomalous signature. This nearly void site could be attributed to the absence of a neighbour OprN molecule in the packing, and thus facing the four-fold axis of the tetragonal space group and the huge cavity built around that axis. In the two former S3 sites (S3a and S3b), the Leu 149 lateral chain is rotated out of its position as compared to the native OprN-wt structure, a feature rarely observed in xenon cavities, which always show equivalent side-chain orientations, with or without xenon.

Finally, a last xenon atom was observed in a cavity carved in the inner bank of the α-domain, at the level of the buoy domain, in a hydrophobic zone of the tunnel (see Fig 2A and S5 Fig).

### The xenon binding sites in the β-barrel domain

Seven more xenon atoms are observed around the membrane β-barrel in the highly hydrophobic zone corresponding to the outer membrane bilayer (Fig 2A). These xenon atoms form a chain of discrete well-characterized sites, as observed in the S4 Fig, together with 2Fo-Fc electron density maps.

### Hydropathy profile of the tunnel

**Fig 4:**
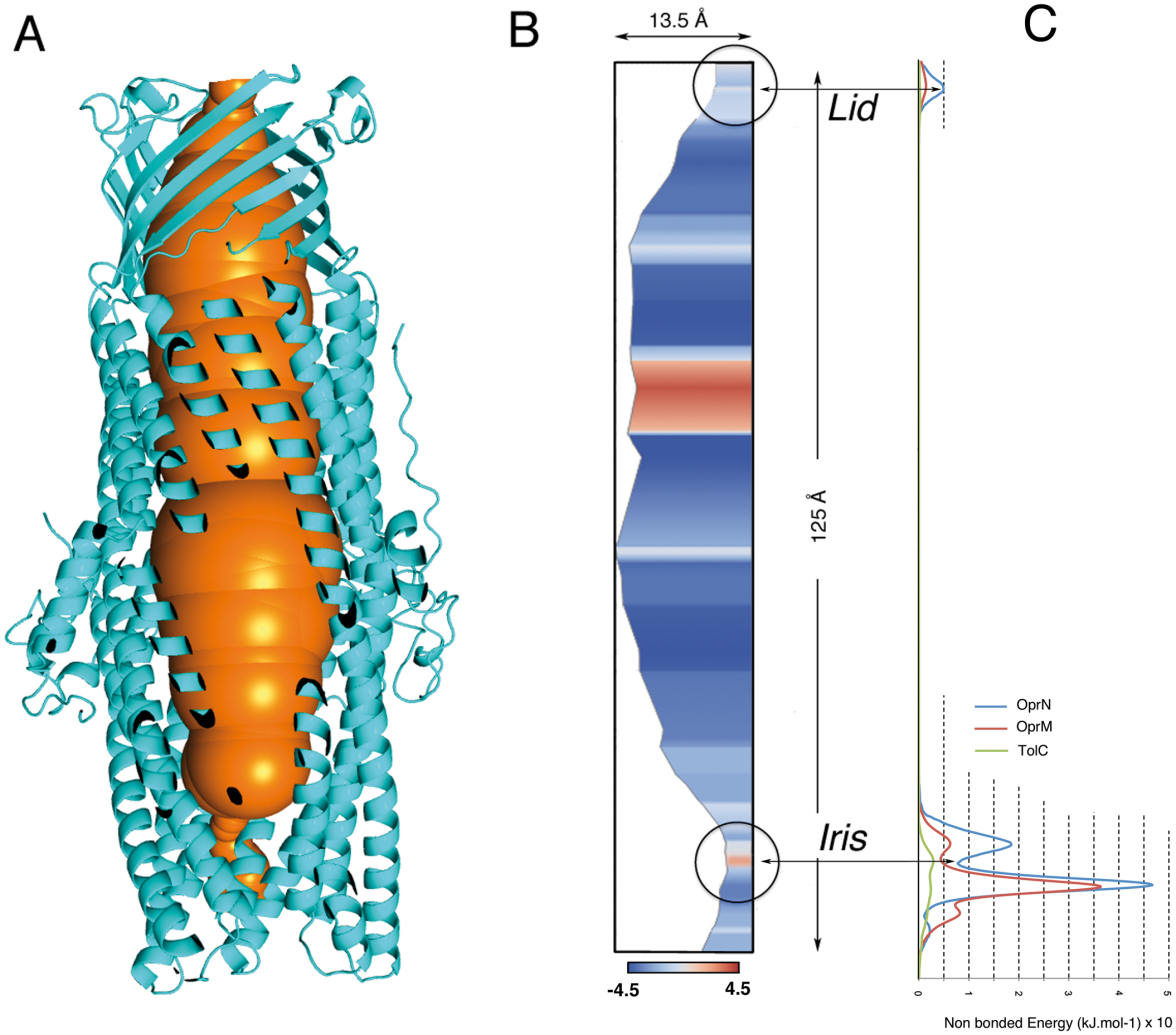
**A:** The huge central cavity of OprN constricted at both ends, towards the periplasm (bottom), and the exterior of the cell (top). **B:** At the same scale, the cavity's profile as seen by the ChExVis program [35] with the two constricted regions pointed by the two red arrows. The gradient profile of the cavity is given as the standard Kyte & Doolittle scale of polar/hydrophobic amino-acids (color code is blue to red for increasing hydropathy). **C:** Non-bonded energy calculated for a xenon atom rolling along the central tunnel of OprN, superposed to the same calculations with OprM and TolC structures (3d5K and 1ek9 from PDB). OprN, OprM, and TolC traces are in blue, orange and lime colors. Energies are calculated using the Lennard-Jones parameters from the CHARMM22 forcefield [34].

As proteins/Xenon interactions are closely linked to hydrophobic notions, it appeared important to explore the hydrophobicity variation along the inner face of the tunnel. The hydropathy profile was visualized by the ChExVis program [35] (Fig 4B), and the non-bonded energy for a xenon was calculated by rolling the atom along the tunnel axis (Fig 4C).

Two hydrophobic regions of the tunnel are highlighted in the ChExVis diagram of the cavity. The first hydrophobic region is localized at the periplasmic extremity (the M1 xenon site previously described). This constricted region is built by a number of conserved residues arranged in specific polar/hydrophobic/polar rings, stacked on top of each other’s. This arrangement induces a strong gradient of polarity along the central channel and on a very short distance. the iris-like closed structure corresponds to a minimum energy at the Leucine-405 triplet, in sandwich between the two high energy barriers of Asp-409 / Arg-412 on one side, and Asp-403 / Arg-396 on the other side. It is worth noting that an equivalent profile is obtained from the OprM tunnel crossing but not for TolC, in which the channel entrance is wider.

The second and larger hydrophobic zone is located at the junction of the β-barrel domain and the periplasmic α-domain and built by three hydrophobic residues, Leu 70, Val 74, and Leu 134, pointing inward the cavity. Associated three by three, they construct a cylinder about 18 to 20 Å in diameter and 8 Å long leading to a sharp change in the overall polarity along the tunnel at this level. Equivalent hydropathy changes can also be observed in other OMF's like TolC or OprM but they are differently distributed along their tunnel axis, pointing out differences that could be associated to a selectivity for a given class of drugs, as experimentally observed [3, 8]. The role of this particular hydrophobic patch is not obvious; it should be implicated in triggering the concerted motions of the helical domains in the overall dynamic of the open/close motions leading to the drug efflux, a motion recently investigated by normal mode analyses on the homologous OprM [13]. A xenon site was observed at this level (Xe-507 in S2 Table, S5 Fig).

## Discussion

Noble gases are known to be chemically non-reactive but biochemically active compounds. In particular, xenon has remarkable properties as inhalational clinical anaesthetic [36–38], together with strong neuroprotective properties [39, 40]. Crystallographic studies under pressure have allowed a better understanding of the mechanisms by which these inert gases act toward a large panel of proteins, and especially neuronal targets, as demonstrated on protein models [41], and functional gated ion channels [22]. The remarkable and widely observed interactions of xenon with proteins are now clearly understood as a multi-process perturbation in their catalytic activities. Even if a small and reversible interaction may affect a single localized protein in a multi-component system or in signalling pathways, a large perturbation effect is expected at the end of the cascade after the sum of many individual weak interactions. On the other hand, hydrophobic inner cavities in proteins play key roles in their function [42, 43]. Their flexibility/rigidity allow to optimize the catalytic efficiency by lowering in energy landscapes the Gibbs free energy of their processes [44, 45]. Varying and playing with the thermodynamic parameters (Pressure and Temperature) of the processes is a way to tackle this problem [46, 47]. Interactions of both xenon and cavities are far from being simple processes, and many studies were devoted to untangle these associations [48–50].

Xenon interacts mainly through induced–induced dipolar interactions (London forces, varying as ~1/r^6^) that become rather efficient at short distances when xenon nests in small cavities and develops many interactions toward hydrophobic amino acid side chains around without disturbing too much the overall conformation of the cavity [20]. As such, xenon cannot be considered as a true inhibitor but just as a "troublemaker" able to disturb functional dynamic properties of its target. Simply releasing xenon pressure restores the full activity. Such a behaviour was proposed from kinetic experiments with globular proteins under xenon atmosphere [49] and may be extended to the OprN-Xe complex.

It is now widely admitted that the gated function and the open/close equilibrium of OMF's, and especially OprN, is critically controlled by the two extremities of the 124 Å long tunnel formed upon the molecular trimerization. The main gated region is the iris-like structure located in the periplasm and connected to the RND molecular transporter (Figs 1 and 4). This region corresponds exactly to the main site of xenon, M1, the most important feature observed here in the OprN-Xe structure. It shows uncommon aspects: purely hydrophobic on one side (the Leu 405 ring), and strongly polar on the other side (the circular ring of salt bridges Asp 409 / Arg 412). This region was also described to be responsible of maintaining the closed state of the channel OprM [13] (Figs 3A and 4B and 4C for OprN). Similarly, in TolC, the corresponding and conserved Asp 374 forms a ring that prevents the export when cross-linked [51]. In addition, Asp 374 associated with Asp 371, is also responsible of the cobalt hexamine blockade of the TolC channel [16].

The specific topology of the M1 site, with a thin hydrophobic zone in sandwich between two polar regions, can be considered as an additional driving force expelling the drug out of the cell to the passive efflux mechanism.

Regarding the secondary sites, all located between intra or inter coiled-coil helices responsible of the concerted open/close allostery, they are likely to rigidify the overall coiled-coil structure of the α-domain by introducing additional constrictions when occupied by xenon atoms, a feature previously observed on globular proteins [50]. Here again, because of the lack of an OprN open-state X-ray structure for comparisons, it is difficult to firmly assess this hypothesis.

In addition to the M and S sites all located in the a-domain, seven additional xenon atoms are observed at the level of the β-barrel domain (Fig 3A). They interact directly with the β-strands of the three subunits building the 12-stranded β-barrel locked in the outer membrane, filling hydrophobic cavities built around the β-barrel domain. These sites are indeed not symmetric as compared to the others, and are likely to have no biological significance. They however protect and stabilize the β-barrel of OprN against the external polar medium, a feature already observed in porin's crystals under xenon pressure [52].

## Conclusion

The OprN channel of the drug transporter MexEF-OprN was analysed in the presence of xenon by X-ray diffraction. The present study evidences the key role of the central iris-like bottleneck built by the highly conserved residues Leu 405 on one side, and Asp 409, Arg 412, on the other side, all forming the molecular closing system suspected to control the open/close state of the channel in the periplasm. Xenon highlights a strong discontinuity in the hydrophobic gradient along the channel at this level that might be an additional driving force to the passive mechanism for the drug transport out of the cell. However, this important cavity shows a higher polarity and is known to host cobalt hexamine, characterized as an opening blocker in TolC, another member of the family. Even if xenon cannot be considered as an inhibitor, it is likely to hamper in the specific case of OMF's the two proposed transport mechanisms: the iris-like mechanism on the periplasmic side, by filling this constricted key-cavity along the channel, and the overall concerted stretch/rotation mechanism by filling specific hydrophobic cavities, built by the coiled-coil long helices forming the cylindrical scaffold of the α-domain.

## Supporting information

S1 Table The xenon sites in OprN– α domain (at 20 bar). (PDF)

S2 Table The xenon sites in OprN—β barrel domain (at 20 bar). (PDF)

S1 Fig Structural alignment of different OMFs with OprN. According to ENDscript (Robert, X., Gouet, P., Nucl. Acids. Res. (2014) 42(w1): w320-324). Pdb codes 5IUY, 3DK5, 5AZS, 4MT0, 4MT4, 3PIK and 1YC9 correspond to OMF proteins OprN, OprM, OprJ, MtrE, CmeC, CusC, and VceC, respectively. The amino acid sequence numbering corresponds to OprN. Strictly identical residues are highlighted in red and similar residues in yellow. The buoy domain is represented by the blue boxes. "acc" is the relative residue accessibility: buried in white, intermediate in cyan, and accessible in blue. "hyd" is the hydropathy scale, from hydrophobic in pink to intermediate in grey, and hydrophilic in cyan.
(PDF)

S2 Fig **A**: The His-tag on one monomer at left (blue C chain), the β-OG molecule (yellow C chain), the nickel atom (green sphere) and a xenon (S3 site—pink sphere), are shown. They are observed parallel to the 136–146 helix in the buoy domain, and are responsible of breaking the three-fold symmetry at this level of the trimer. **B**: Crystal packing of OprN in the I4 space group showing the perpendicular interaction of two channels responsible of the non-equivalence observed at site S3.
(PDF)

S3 Fig OprN xenon sites. The 2Fo-Fc maps are drawn at electron density greater than 2.5 σ the map average. (**A**: site M1, **B**: one of the S1 sites at the same level). **C** and **D**: The two secondary S2 and S3 sites in subunit B.
(PDF)

S4 Fig Three xenon sites lining the periphery of the hydrophobic β-barrel domain. The 2Fo-Fc map is contoured at 2.5 σ. Top: site Xe 503B, lower left: Xe 502B and lower right: Xe 501B (see S2 Table).
(PDF)

S5 Fig The xenon atom located at the level of the buoy domain. Located in the wide hydrophobic patch of the tunnel.
(PDF)
