# Supplementary material for: Xenon for tunnelling analysis of the efflux pump component OprN
Source: PLoS One. 2017 Sep 8;12(9):e0184045. doi: 10.1371/journal.pone.0184045 (PMC5590881; doi:10.1371/journal.pone.0184045)
Supplement: S1 Table — (PDF) [file pone.0184045.s001.pdf]

S1 Table **The xenon sites in OprN - α domain (at 20 bar)**

Site Label / subunit	Refined occupancy (%) / height in initial anomalous map (σ)	Location	Distances (Å)…residue	
Xe 503 / A	100 / 31.0	site M1 on axis (iris)	3.94 …CD1 Leu 405 A<br>3.96 …CD2 Leu 405 B<br>3.88 …CD1 Leu 405 C<br>3.63 …OD1 Asp 409 A<br>3.81 …OD1 Asp 409 B	3.66 …OD1 Asp 409 C<br>3.78 …NH1 Arg 412 A<br>4.07 …NH1 Arg 412 B<br>4.09 …NH1 Arg 412 C
Xe 504 / A	30 / 7.0	Site S1 intra-subunit A	3.84 …CA Ala 201 A<br>3.92 …CB Gln 177 A<br>4.11 …CG Arg 204 A<br>4.76 …CD1 Leu 408 A	3.79 …O Ala 201 A<br>3.85 … O Gln 177 A<br>4.50 …N Arg 181 A<br>4.23 … CZ Phe 404 A
Xe 506 / B	30 / 6.3	Site S1 intra-subunit B	3.78 …CB Ala 201 B<br>3.78 …CB Gln 177 B<br>3.99 …CG Arg 204 B<br>3.90 …CB Ser 180 B<br>3.18 …OE1 Glu 411 B	3.58 …O Ala 201 B<br>4.05 …CG Gln 177 B<br>4.46 …N Arg 181 B<br>4.47 … CZ Phe 404 B<br>4.57 …OE2 Glu 411 B
Xe 505 / C	30 / 4.9	Site S1 intra-subunit C	3.79 …CB Ala 201 C<br>3.74 …CB Gln 177 C<br>4.16 …CG Arg 204 C<br>4.23 …CB Ser 180 C<br>3.31 …OE1 Glu 411 C<br>3.47 …O Gln 177 C	3.64 …O Ala 201 C<br>3.95 …CG Gln 177 C<br>4.49 …N Arg 181 C<br>4.19 … CZ Phe 404 C<br>4.45 …CD Glu 411 C
Xe 505 / A	45 / 12.5	Site S2 inter-subunit A/C	3.66 …CA Gln 214 A<br>4.06 …C Pro 213 A<br>3.82 …CB Lys 371 C<br>4.10 …CD Arg 375 C<br>4.19 …CB Ala 217 A	3.49 …OE1 Gln 214 A<br>3.92 …O Pro 213 A<br>3.67 …CG Arg 372 C<br>4.37 …CB Arg 375 C<br>3.32 …O HOH 683 C
Xe 508 / A	35 / 9.5	Site S2 inter-subunit A/B	3.59 …CA Gln 214 B<br>4.22 …C Pro 213 B<br>4.22 …CB Lys 371 A<br>3.99 …CD Arg 375 A<br>4.25 …CB Ala 217 B	3.49 …OE1 Gln 214 A<br>3.92 …O Pro 213 A<br>3.67 …CG Arg 372 C<br>4.45 …CB Arg 375 A<br>3.38 …O HOH 654 A
Xe 509 / B	40 / 9.0	Site S2 inter-subunit B/C	3.65 …CA Gln 214 C<br>4.03 …CB Gln 214 C<br>4.25 …C Pro 213 C<br>4.10 …CB Lys 371 B<br>4.00 …CD Arg 375 B<br>4.29 …CB Ala 217 C<br>4.01 …C Lys 371 B	4.00 …CG Gln 214 C<br>4.30 …OE1 Gln 214 C<br>4.13 …O Pro 213 C<br>3.69 …CG Arg 372 B<br>4.35 …CB Arg 375 B<br>3.38 …O HOH 689 B<br>3.72 …N Arg 372 B
Xe 507 / B	25 / 7.5	Site S3 intra-subunit B	3.17 …CG Leu 149 B<br>3.82 …CD2 Leu 51 B<br>4.31 …CD2 Leu 57 B<br>2.95 …CD1 Leu 149 B<br>4.37 …N Ile 150 B	4.10 …CB Ser 50 B<br>3.41 …OG Ser 50 B<br>4.08 …O Ser 50 B<br>4.73 …CG1 Ile 150 B<br>2.94 …CB Leu 149 B
Xe 506 / C	23 / 7.6	Site S3 intra-subunit C	3.67 …CG Leu 149 C<br>4.12 …CD2 Leu 51 C<br>4.12 …CD1 Leu 57 C<br>3.78 …CD1 Leu 149 C<br>4.30 …N Ile 150 C<br>4.23 …O Ser 50 C<br>4.53 …CG Asn 54 C	4.08 …CB Ser 50 C<br>3.23 …OG Ser 50 C<br>4.20 …CG Leu 57 C<br>4.73 …CG1 Ile 150 C<br>3.09 …CB Leu 149 C<br>4.24 …CB Asn 54 C<br>4.33 …C Ser 50 C
