# Supplementary material for: Xenon for tunnelling analysis of the efflux pump component OprN
Source: PLoS One. 2017 Sep 8;12(9):e0184045. doi: 10.1371/journal.pone.0184045 (PMC5590881; doi:10.1371/journal.pone.0184045)
Supplement: S2 Table — (PDF) [file pone.0184045.s002.pdf]

S2 Table **The xenon sites in OprN - β barrel domain**

Site Label / subunit	Refined occupancy (%) / height in initial anomalous map (σ)	Location	Distances (Å)…residue	
Xe 506 / A	35 / 5.7	β-domain, inter-subunit A/C	4.30 …CB Ser 84 A<br>4.15 …CA Arg 85 A<br>4.61 …CB Ala 86 A<br>4.27 … CG1 Ile 323 C	4.56 …CB Trp 325 C<br>4.07 …O Ile 323 C<br>3.61 …OG Ser 84 A
Xe 507 / A	20 / 4.9	β-domain, intra-subunit A	4.09 …CB Leu 299 A<br>4.07 …CB Ser 114 A<br>5.09 …CG1 Val 82 A	4.75 …N Leu 299 A<br>4.60 …OG Ser 114 A
Xe 501 / B	45 / 9.8	β-domain, intra-subunit B	4.32 …CG1 Ile 309 B<br>4.62 …CD2 Tyr 108 B<br>4.10 …CB Leu 110 B<br>4.03 …CB Ala 86 B<br>4.59 …Xe 502 B	4.51 …CG2 Ile 309 B<br>4.70 …CB Tyr 108 B<br>4.69 …CD1 Leu 110 B<br>4.34 …CA Ala 86 B
Xe 502 / B	35 / 8.1	β-domain, inter-subunit A/B	4.39 …CD1 Ile 323 A<br>4.48 …C Pro 321 A<br>4.65 …CG Pro 321 A<br>4.19 …CA Ser 87 B	4.35 …CG1 Ile 323 A<br>4.47 …CB Pro 321 A<br>4.33 …CB Ala 86 B<br>4.59 … Xe 501 B
Xe 503 / B	35 / 5.3	β-domain, inter-subunit A/B	4.56 …CB Ala 86 B<br>4.42 …C Arg 85 B<br>4.31 …N Arg 85 B<br>4.45 …CB Ser 84B	4.50 …CG2 Ile 323 A<br>4.52 …CA Arg 85 B<br>4.51 …C Ser 84 B<br>4.49 …CB Trp 325 A
Xe 501 / C	22 / 6.4	β-domain, intra-subunit C	4.29 …CG1 Val 293 C<br>4.48 …CD1 Leu 295 C<br>3.99 …CB Plm 501 A *	4.49 …CD2 Leu 118 C<br>4.38 …CB Pro 321 C
Xe 507 / C	25 / 5.5	tunnel [a]	3.75 …CG Arg 225 C<br>4.47 …CD Gln 159 C<br>4.18 …CA Ala 222 C	4.26 …CB Arg 225 C<br>4.42 …CB Ala 222 C<br>3.15 …O HOH 761 C
Xe 508/ C	20 / 3.7	β-domain, inter-subunit B/C	4.41 …CB Trp 325 B<br>4.53 …CG2 Ile 323 B<br>4.52 …C Arg 85 C<br>4.41 …CB Ser 84 C	4.68 …CC PLM 504 B<br>4.60 …CB Ala 86 C<br>4.60 …CA Arg 85 C<br>3.62 …OG Ser 84 C

[a] This xenon is located on the inner bank of the tunnel, at the level of the buoy domain.
