# Supplementary material for: Xenon for tunnelling analysis of the efflux pump component OprN
Source: PLoS One. 2017 Sep 8;12(9):e0184045. doi: 10.1371/journal.pone.0184045 (PMC5590881; doi:10.1371/journal.pone.0184045)

S1 Fig **Structural alignment of different OMFs with OprN** According to ENDscript (Robert, X., Gouet, P., Nucl. Acids. Res. (2014) 42(w1): w320-324). Pdb codes 5IUY, 3DK5, 5AZS, 4MT0, 4MT4, 3PIK and 1YC9 correspond to OMF proteins OprN, OprM, OprJ, MtrE, CmeC, CusC, and VceC, respectively. The amino acid sequence numbering corresponds to OprN. Strictly identical residues are highlighted in red and similar residues in yellow. The buoy domain is represented by the blue boxes. "*acc*" is the relative residue accessibility: buried in white, intermediate in cyan, and accessible in blue. "*hyd*" is the hydropathy scale, from hydrophobic in pink to intermediate in grey, and hydrophilic in cyan.

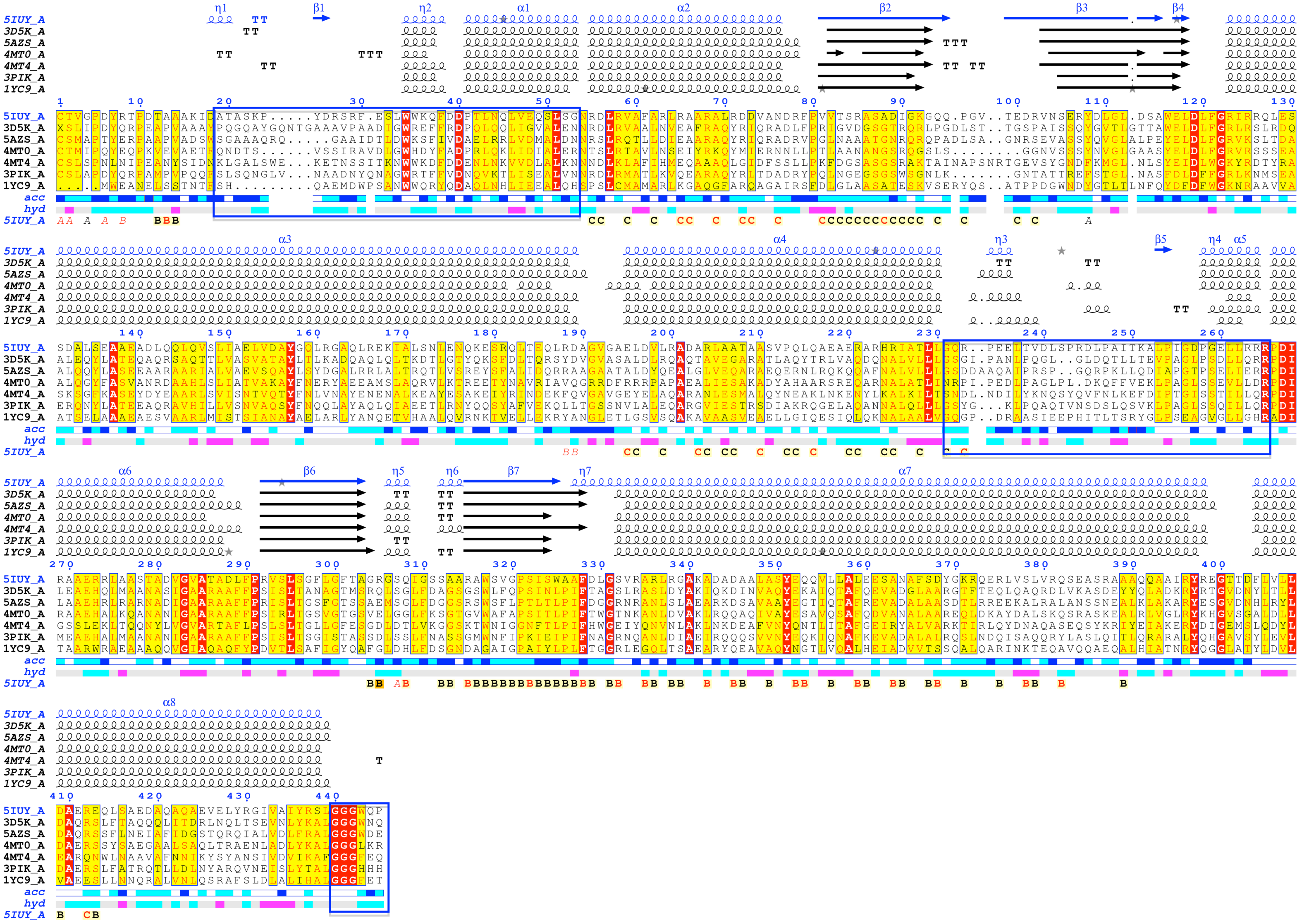

Supplement: S1 Fig — According to ENDscript (Robert, X., Gouet, P., Nucl. Acids. Res. (2014) 42(w1): w320-324). Pdb codes 5IUY, 3DK5, 5AZS, 4MT0, 4MT4, 3PIK and 1YC9 correspond to OMF proteins OprN, OprM, OprJ, MtrE, CmeC, CusC, and VceC, respectively. The amino acid sequence numbering corresponds to OprN. Strictly identical residues are highlighted in red and similar residues in yellow. The buoy domain is represented by the blue boxes. "acc" is the relative residue accessibility: buried in white, intermediate in cyan, and accessible in blue. "hyd" is the hydropathy scale, from hydrophobic in pink to intermediate in grey, and hydrophilic in cyan. (PDF) [file pone.0184045.s003.pdf]
