# Supplementary material for: Xenon for tunnelling analysis of the efflux pump component OprN
Source: PLoS One. 2017 Sep 8;12(9):e0184045. doi: 10.1371/journal.pone.0184045 (PMC5590881; doi:10.1371/journal.pone.0184045)

S2 Fig **A**: The His-tag on one monomer at left (blue C chain), the β–OG molecule (yellow C chain), the nickel atom (green sphere) and a xenon (S3 site - pink sphere), are shown. They are observed parallel to the 136-146 helix in the buoy domain, and are responsible of breaking the three-fold symmetry at this level of the trimer. **B**: Crystal packing of OprN in the I4 space group showing the perpendicular interaction of two channels responsible of the non-equivalence observed at site S3.

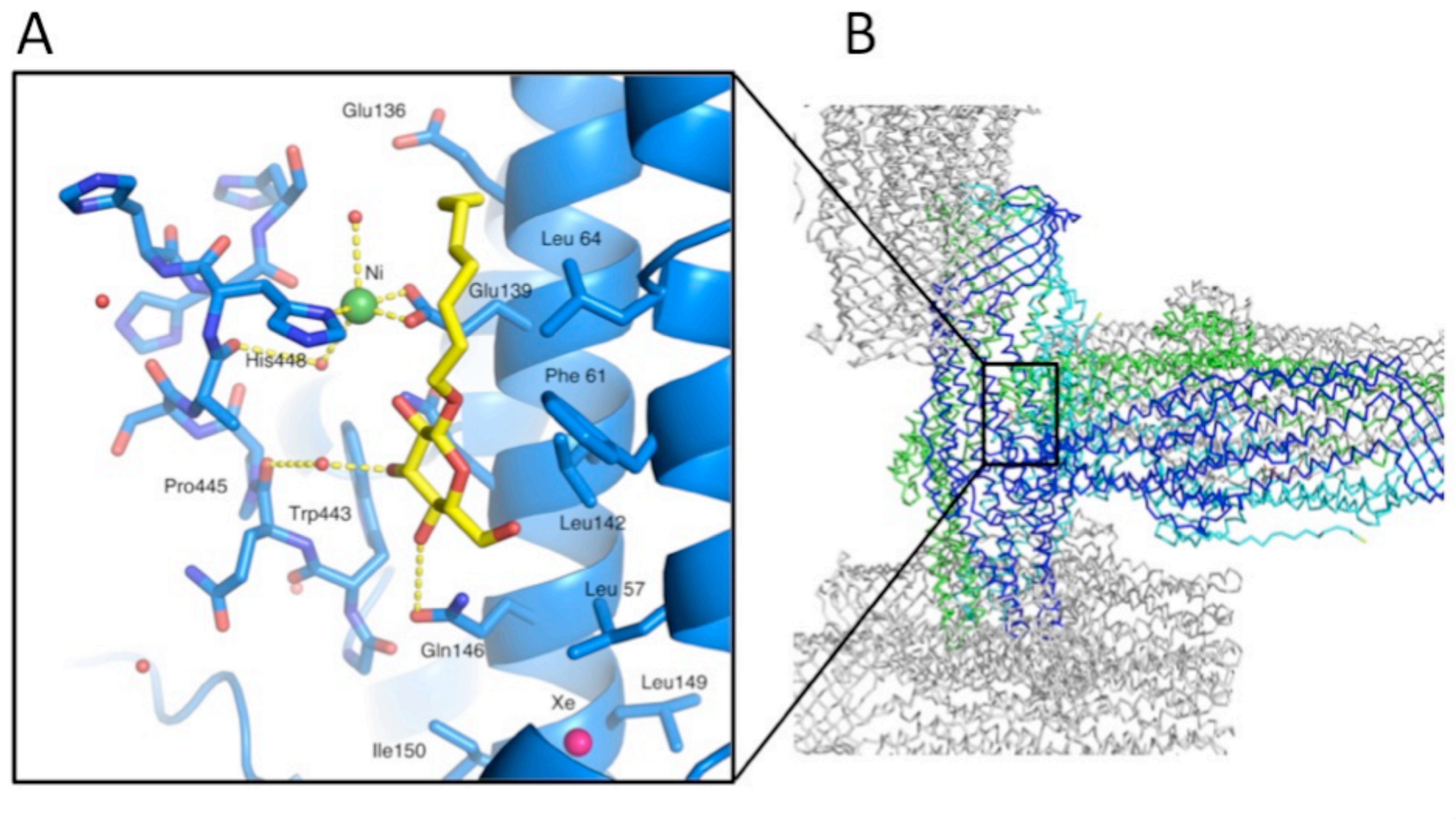

Supplement: S2 Fig — A: The His-tag on one monomer at left (blue C chain), the β-OG molecule (yellow C chain), the nickel atom (green sphere) and a xenon (S3 site—pink sphere), are shown. They are observed parallel to the 136–146 helix in the buoy domain, and are responsible of breaking the three-fold symmetry at this level of the trimer. B: Crystal packing of OprN in the I4 space group showing the perpendicular interaction of two channels responsible of the non-equivalence observed at site S3. (PDF) [file pone.0184045.s004.pdf]
