# Supplementary material for: Xenon for tunnelling analysis of the efflux pump component OprN
Source: PLoS One. 2017 Sep 8;12(9):e0184045. doi: 10.1371/journal.pone.0184045 (PMC5590881; doi:10.1371/journal.pone.0184045)

S3 Fig **OprN xenon sites**. The 2Fo-Fc maps are drawn at electron density greater than 2.5 σ the map average. (**A**: site M1, **B**: one of the S1 sites at the same level). **C** and **D**: The two secondary S2 and S3 sites in subunit B.

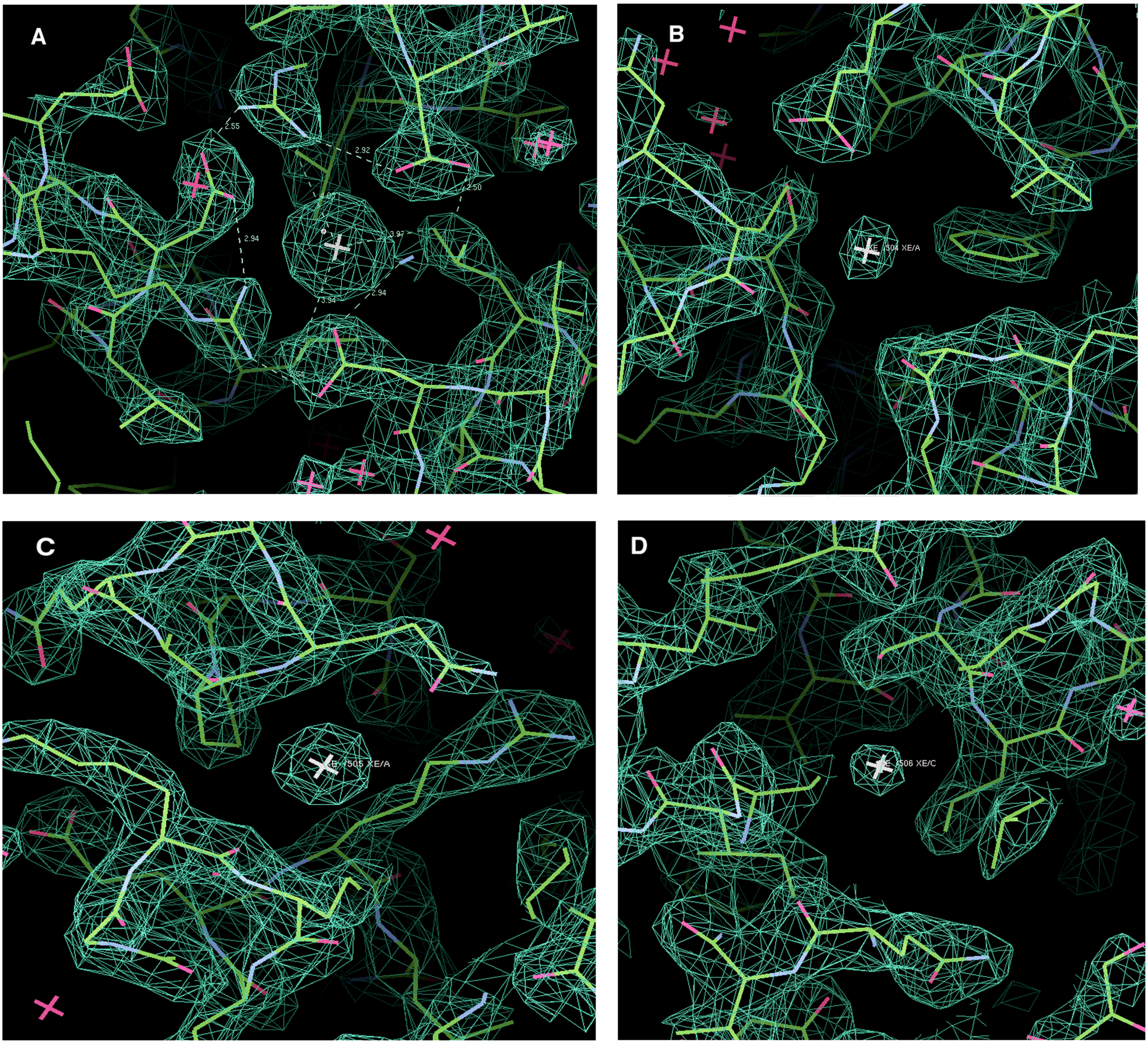

Supplement: S3 Fig — The 2Fo-Fc maps are drawn at electron density greater than 2.5 σ the map average. (A: site M1, B: one of the S1 sites at the same level). C and D: The two secondary S2 and S3 sites in subunit B. (PDF) [file pone.0184045.s005.pdf]
