# Supplementary material for: Xenon for tunnelling analysis of the efflux pump component OprN
Source: PLoS One. 2017 Sep 8;12(9):e0184045. doi: 10.1371/journal.pone.0184045 (PMC5590881; doi:10.1371/journal.pone.0184045)

S4 Fig **Three xenon sites lining the periphery of the hydrophobic β-barrel domain**. The 2Fo-Fc map is contoured at 2.5 σ. Top: site Xe 503B, lower left: Xe 502B and lower right: Xe 501B (see Table S2).

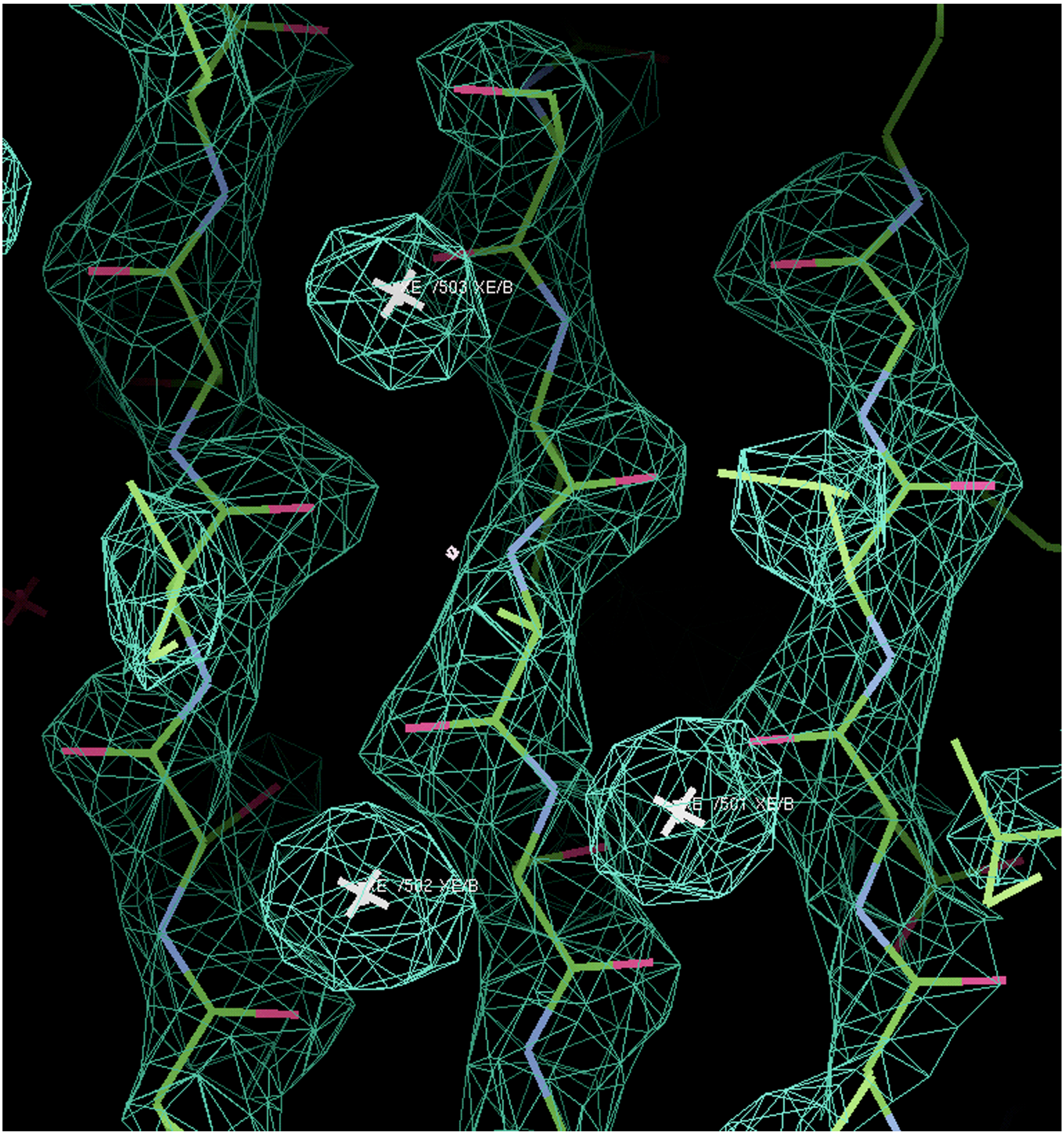

Supplement: S4 Fig — The 2Fo-Fc map is contoured at 2.5 σ. Top: site Xe 503B, lower left: Xe 502B and lower right: Xe 501B (see S2 Table). (PDF) [file pone.0184045.s006.pdf]
