# Supplementary material for: Xenon for tunnelling analysis of the efflux pump component OprN
Source: PLoS One. 2017 Sep 8;12(9):e0184045. doi: 10.1371/journal.pone.0184045 (PMC5590881; doi:10.1371/journal.pone.0184045)

S5 Fig **The xenon atom located at the level of the buoy domain**. Located in the wide hydrophobic patch of the tunnel.

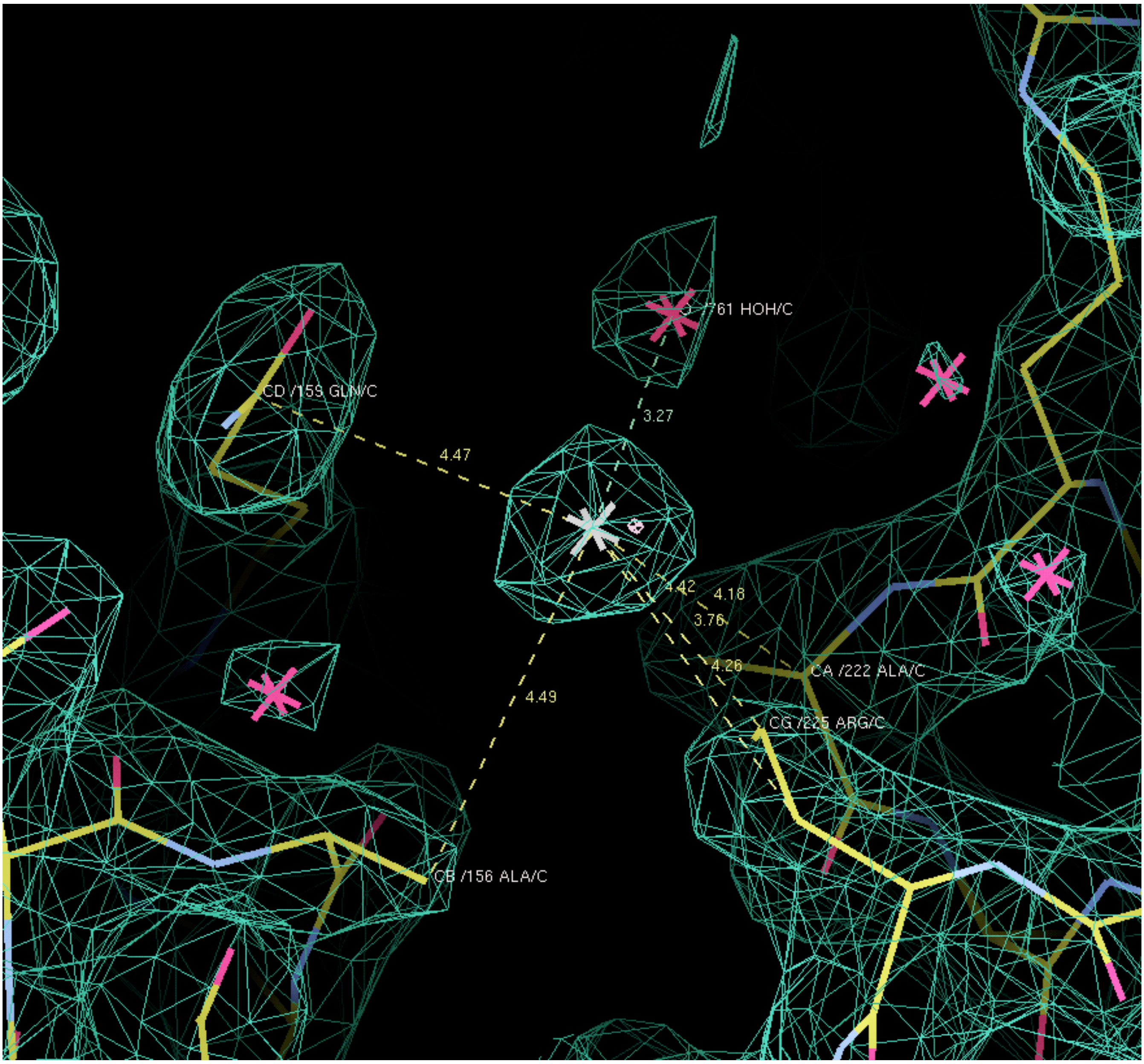

Supplement: S5 Fig — Located in the wide hydrophobic patch of the tunnel. (PDF) [file pone.0184045.s007.pdf]
